# The COMTval158met polymorphism is associated with symptom relief during exposure-based cognitive-behavioral treatment in panic disorder

**DOI:** 10.1186/1471-244X-10-99

**Published:** 2010-11-26

**Authors:** Tina B Lonsdorf, Christian Rück, Jan Bergström, Gerhard Andersson, Arne Öhman, Nils Lindefors, Martin Schalling

**Affiliations:** 1 Psychology section, Karolinska Institutet, Fogdevreten 2a, 17176 Stockholm, Sweden; 2 Division of Psychiatry, Karolinska University Hospital Huddinge, 141 86 Stockholm, Sweden; 3 Neurogenetics Unit, Center for Molecular Medicine, Karolinska Institutet Karolinska Hospital, L8:00, 171 76 Stockholm, Sweden; 4 Stockholm Brain Institute, Stockholm, Sweden; 5 Nordic center of Excellence in Cognitive Control, Stockholm, Sweden; 6 Department of Behavioural Sciences and Learning, Linköping University, Campus Valla, House I:3, 581 83 Linköping, Sweden; 7 Institute for Systems Neuroscience, University Hospital Hamburg-Eppendorf, Martinistrasse 52 (W34), Martinistr. 52, 20251 Hamburg, Germany

## Abstract

**Background:**

Cognitive behavioral therapy (CBT) represents a learning process leading to symptom relief and resulting in long-term changes in behavior. CBT for panic disorder is based on exposure and exposure-based processes can be studied in the laboratory as extinction of experimentally acquired fear responses. We have recently demonstrated that the ability to extinguish learned fear responses is associated with a functional genetic polymorphism (COMTval158met) in the *COMT *gene and this study was aimed at transferring the experimental results on the COMTval158met polymorphism on extinction into a clinical setting.

**Methods:**

We tested a possible effect of the COMTval158met polymorphism on the efficacy of CBT, in particular exposure-based treatment modules, in a sample of 69 panic disorder patients.

**Results:**

We present evidence that panic patients with the COMTval158met met/met genotype may profit less from (exposure-based) CBT treatment methods as compared to patients carrying at least one val-allele. No association was found with the 5-HTTLPR/rs25531 genotypes which is presented as additional material.

**Conclusions:**

We were thus able to transfer findings on the effect of the COMTval158met polymorphism from an experimental extinction study obtained using healthy subjects to a clinical setting. Furthermore patients carrying a COMT val-allele tend to report more anxiety and more depression symptoms as compared to those with the met/met genotype. Limitations of the study as well as possible clinical implications are discussed.

**Trial registration:**

Clinical Trial Registry name: Internet-Versus Group-Administered Cognitive Behavior Therapy for Panic Disorder (IP2). Registration Identification number: NCT00845260, http://www.clinicaltrials.gov/ct2/show/NCT00845260

## Background

Cognitive behavioral therapy (CBT) represents a learning process leading to symptom relief. It results in long-term changes in behavior, which have their correlates in altered neural activity [for a review see e.g., [1]], gene expression as well as synaptic connectivity in the brain [2]. CBT for anxiety disorders generally, and panic disorder (PD) specifically, is based on exposure. These exposure-based processes can be studied in the laboratory as the extinction of experimentally acquired fear responses. We have recently reported that the ability to extinguish learned fear responses is associated with a functional polymorphism (COMTval158met) in the Catechol-O-methyltransferase (*COMT*) gene [3].

COMT is a methylation enzyme metabolizing monoaminergic neurotransmitters including dopamine [4]. The COMTval158met polymorphism is an A/G single-nucleotide polymorphism (SNP) causing an amino acid exchange from valine (val) to methionine (met) at codon 158. The val-allele is associated with a three- to four-fold higher COMT activity as compared to the thermolabile met-allele [5,6].

The met/met genotype has been associated with resistance to extinction [3] and higher severity of PTSD even at low traumatic load [7]. Furthermore, the met-allele has been associated with negative emotionality, altered prefrontal cortex activity [for a review see e.g. [8]] and has been suggested to be associated with reduced cognitive flexibility but increased cognitive stability [9]. Given the cognitive change induced by therapy and the pivotal role of prefrontal brain areas in extinction, the met-allele might also be associated with the efficacy of CBT in PD.

The val-allele of the COMTval158met polymorphism has been associated with PD in several case-control studies [e.g., [10,11]] and a recent meta-analysis supports an association of the COMT val-allele with the diagnosis of PD in Caucasians [12], despite of contradictory findings.

This study aimed at transfering the experimental results of the COMTval158met polymorphism on extinction into a clinical setting by investigating the efficacy of CBT (in particular exposure-based treatment modules) in a sample of PD patients, genotyped for the COMTval158met.

## Methods

### Patient population

Patients were recruited from a randomized clinical equivalence trial (CET) of regular cognitive-behavioral group therapy (gCBT) vs. internet-based CBT (iCBT) for PD [13](see additional file 1). At the time of inclusion to the CET, patients that consented also to participate in clinical genetic studies donated a blood sample. Inclusion criteria were: (1) fulfilling DSM-IV criteria for PD with/without agoraphobia (PD/A), (2) PD/A primary diagnosis, (3) non-physiological etiology of panic symptoms, (4) age at least 18 years, (5) not suffering from severe depression or suicidal ideation, (6) if taking prescribed drugs for panic disorder, having had a constant dosage for 2 months prior to inclusion (7) not undergoing other CBT or psychotherapy.

Eighty-seven patients consented to participate in the study. Fourteen patients were excluded because of excessive missing data (e.g drop-out right after inclusion before beginning CBT), three patients were excluded because of questionable clinical significance of their PD at the time of inclusion, and one patient was excluded due to non-Caucasian ancestry. This left us with a final sample of 69 Caucasian patients (see Additional file 1 Figure S1), with 26 male (mean = 32.8 years, SD = 7.6, range 22-53) and 43 female (mean = 36.9 years, SD = 10.3, range 23-61) patients. The majority (n = 39) of the patients were referred from primary health care, 25 by self-referral and five by psychiatric outpatient care units. Thirty patients were pharmacologically treated (SSRIs, other antidepressants, Benzodiazepine) at assignment to the study, with a stable dosage for at least 2 months. Hence, symptoms were still present in this group in spite of medication. Patients were allowed to continue pharmacological treatment during CBT. All patients provided written informed consent and the study was approved by the Regional Ethics Committee in Stockholm and was carried out in accordance with the Declaration of Helsinki.

### Diagnostic procedure

A psychiatrist or a supervised resident in psychiatry performed an in-person structured clinical interview including the Mini-International Neuropsychiatric Interview (M.I.N.I. [14]). The diagnostic procedures were performed blind to genotype and group assignment (gCBT vs ICBT) in the treatment trial.

After each of the ten modules of CBT treatment, patients filled in the Hospital Anxiety and Depression Scale [HADS, [15]] which comprises an anxiety subscale (7 items) and a depression subscale (7 items). All items are rated on a 4-point likert scale. Overall, the subscales have good psychometric properties [16]. We used the HADS as it measures symptoms of anxiety and depression in a discrete way without much effort needed from the patient.

### Treatment

Patients were randomly assigned to one of two treatment arms: regular group CBT (gCBT, N = 38) or internet-based CBT (ICBT, N = 31). Both treatments lasted for ten weeks and the content was similar, with the exception of the format. The treatment program consisted of ten modules (M), all are based on well established CBT-principles: psycho-education (M1), cognitive restructuring (M2-M3), interoceptive exposure (M4-M5), in vivo exposure (for agoraphobic situations, M6-M9) and relapse prevention (M10). In the ICBT arm, a text-based manual [17] was administered via a web-page with support via e-mail. In the gCBT arm, the manual was administered in print-out form, as well as presented by 2 psychologists. The 10 modules consisted of text as well as exercises, to be performed in the patient's every-day life. In the ICBT arm, access to the next module was not provided before the psychologist had received the answers sent by way of the interactive forms. A psychologist read and answered the communication manually. In the gCBT arm, the group met with the two psychologists during weekly two-hour sessions. Both treatment groups (gCBT vs. ICBT) did not differ in their treatment outcome [13].

### Genotyping

DNA was extracted from whole blood [18] and genotyping for COMTval158met was performed as described in detail earlier [3]. Briefly, we used the Taqman^® ^allelic discrimination assay (5' nuclease assay,[19]) performed on an ABI HT7900 (Applied Biosystems, Foster City, CA).

Table 1 displays the distribution of variables of interest between the different genotype groups (val-carriers [val/val and val/met] vs. met/met).

**Table 1:** Descriptives for COMTval158met genotype groups.

COMTval158met	Val/val and val/met	Met/met	X^2 ^p-value^a^	Mann-Whitney U-test p-value
N	40	29		
sex (M/F)	15/25	11/18	0.97	-
mean age (SD)	37.3 (10.9)	32.7 (6.6)	-	0.11
age of onset (SEM)	27.9 (10.7)	25.3 (8.6)	-	0.39
duration of illness (SEM)	9.8 (11.9)	7.2 (6.4)	-	0.80
Depresssion (yes/no)	9/31	1/28	0.03*	-
iCBT/gCBT	14/26	17/12	0.052	-
Number of modules	6.93 (2.1)	6.31 (2.8)	0.30	
Co-medication^b ^(yes/no)	16/24	14/15	0.50	

^a^: chi2 (df = 1)

^b^: any medication (including SSRI, Benzodiazepine, tricyclic antidepressants)

iCBT: internet-based CBT; gCBT: group CBT

Additional genotyping procedures (for 5-HTTLPR/rs25531) are described as additional methods (see additional file 2) accompanying additional analyses and results (see additional file 3) discussed in the discussion section.

### Statistical analysis

Statistical analysis were performed using SPSS for Windows v15 (SPSS Inc., Chicago, IL, USA) and graphs were made using Origin^®^8 (OriginLab^®^, Northampton, MA, USA). Val-carriers (val/val + val/met) were merged to a val-carrier group *a priori *[3]. However, for means of completeness and in order to facilitate comparability between different studies, we also report results from analyses comparing all three COMTval158met genotypes (val/val, val/met, met/met) as additional material (see additional file 4) as well as results based on the bi-and triallelic 5-HTTLPR (see additional files 2 and 3).

Normal distribution of variables was tested using Shapiro-Wilk's test. Non-parametric tests were used when data significantly differed from the normal distribution (which was the case for the HADS post-treatment anxiety and the depression subscale and all difference scores except for the one mentioned above), otherwise parametric tests were applied (in case of the HADS pre-treatment anxiety and the depression scale and the cognitive block-exposure block difference score for the HADS depression scale, see above for details).

Due to a considerable amount of missing HADS measurements after each module (in particular after M8 and M9), we were unable to perform repeated measures ANOVAs for the 9 measurements during treatment as SPSS excludes cases list-wise.

Thus we combined modules with a similar content to blocks of modules and calculated the mean HADS score of the Modules included in each block. The mean HADS ratings after Module M1-M3 formed a *cognitive block *and the mean HADS scores for Module M4-M9 were grouped into an *exposure block*. This was done for both the anxiety and depression subscale.

As we were particularly interested in a differential response to exposure-based modules, we calculated difference scores between the cognitive and the exposure block [mean cognitive-block - mean exposure-block] as well as differences between the pre-treatment HADS rating and the cognitive block [mean pretreatment - mean cognitive-block]. Difference scores were compared between genotype groups by ANOVAS with difference score as the dependent and COMTval158met genotype as the independent variable.

Partial Eta^2 ^is reported as measurements of effect size. We report results corrected for multiple comparisons by the Bonferroni correction. HWE was tested using the Pearson's goodness-of-fit χ^2 ^(df = 1) provided on a website by http://ihg2.helmholtz-muenchen.de/cgi-bin/hw/hwa1.pl.

## Results

### Patient characteristics and genotype frequencies

COMT genotype frequencies differed significantly from HWE (N = 18 val/val, N = 22 val/met, N = 25 met/met), Pearson's goodness-of fit chi-Square (df = 1), chi2 = 8.25, p < 0.01. The genotype groups (val-carriers [val/val and val/met] vs. met/met) did not differ significantly from each other in any descriptive or clinical variable except for frequency of comorbidity with mild major depressive episode (see Table 1). Patients with the COMT met/met genotype had a lower incidence of comorbidity with depression as compared to val-carriers. We have not included this variable as a covariate in our analyses as this would be questionable in the light of preexisting differences between non-randomized groups (see limitations section in the discussion). Still, when depression was included as a covariate in our primary analyses, results did not change substantially (see below).

Importantly, the number of patients using psychopharmaceuticals in addition to CBT treatment did not differ between the two COMT val158met genotype groups (Table 1) or between the two treatment arms, x^2 ^(df = 1) = 0.055, p = 0.82. The female:male ratio did not differ between the genotype groups (see Table 1) nor between the two treatment arms, x^2 ^(df = 1) = 0.12, p = 0.73. Thus, it was considered unnecessary to include these variables (co-medication and sex) as covariates.

### Symptomatic profile prior to CBT treatment

Univariate ANOVASs with genotype as the independent and mean HADS pre-treatment scores (separate for the two subscales) as the dependent variables revealed, that patients carrying at least one COMT158val allele reported tendentially more symptoms as compared to patients with the met/met genotype in the anxiety subscale of the HADS, F(1,66) = 3.73, p = 0.058, pη^2 ^= 0.05, and significantly more symptoms in the depression subscale of the HADS F(1,66) = 6.33, p = 0.014, pη^2 ^= 0.09.

### Symptomatic profile after treatment

Mann-Whitney U-tests with genotype as the group variable and mean HADS post-treatment scores (separate for the two subscales) as the test variables revealed, no significant differences between the COMTval158met genotype groups on the HADS anxiety scale, p = 0.46. However, patients with the val-allele reported significantly more symptoms on the depression subscale, p = 0.04, even after treatment.

### Symptom relief during the course of treatment

#### Difference score pre-treatment - cognitive block

An univariate ANOVA with the difference score between the mean HADS anxiety score prior to treatment and during the cognitive block as the dependent and COMT158met genotype as the independent variable revealed no significant effect of COMTval158met genotype on this difference score, F(1,62) < 1. No effect was found for the HADS depression scale during the course of treatment using the same analysis as described above either, F(1,62) < 1.1.

#### Difference score cognitive block - exposure block

**Figure 1:**
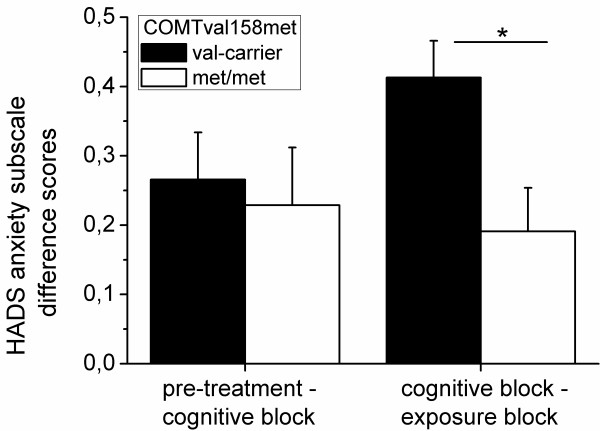
**Difference scores between the HADS anxiety mean scores pre-treatment and during the cognitive block as well as during the cognitive vs. the exposure block for COMT 158val-carriers (black bars) and patients with the met/met genotype (white bars)**.

A univariate ANOVA with the difference score between the mean HADS anxiety score of the cognitive and the mean HADS anxiety score of the expose block as the dependent, COMT158met genotype (met/met vs. val-carriers) as the independent variable revealed a significant effect of COMTval158met genotype, F(1,58) = 5.40, p = 0.024, pη^2 ^= 0.09. Patients with the met/met genotype showed significantly less symptom relief during the exposure block as compared to COMT 158val-carriers (see Figure 1).

An additional analysis including medication status (yes/no), age, sex, pre-treatment HADS anxiety score and the type of treatment (ICBT vs. gCBT) and comorbidity with depression as covariates in the analysis on the difference score between the cognitive and the exposure block revealed no significant impact of these variables. Importantly, the effect of COMTval158met genotype on the outcome measure still remained statistically significant when controlling for all these variables, F(1,52) = 4.73, p = 0.034, pη^2 ^= 0.08.

However, sex had a close to significant impact on the difference score between the cognitive and the exposure block, F(1,52) = 3.96, p = 0.052, pη^2 ^= 0.07. Women had higher difference scores and thus more symptoms relieve than men. Importantly, additional analyses did not yield evidence for a sex × COMTval158met genotype interaction on the difference score between the cognitive and the exposure block, F(1,56) < 1.

A Mann-Whitney-U test revealed no difference between the COMTval158met genotype groups for the HADS depression scale during the course of treatment using difference scores as described above, p = 0.16.

## Discussion

We present first evidence that PD patients with the COMTval158met met/met genotype may profit less from exposure-based CBT treatment methods as compared to patients carrying at least one val-allele (val/val + val/met). We were thus able to transfere findings of the association of the COMTval158met polymorphism from an experimental extinction study [3] obtained using healthy subjects to a clinical setting. Furthermore, our finding of (tendentially) more severe symptom severity in patients with the COMT val-allele (prior to CBT treatment) is in line with studies showing an association of the COMT val-allele with the diagnosis of PD in Caucasians [12]. As we observed tendentially more symptoms of anxiety and anhedonia in val-carriers prior to CBT but no differences in symptom severity after 10-weeks of CBT, this supports our finding of differential efficacy of CBT depending on the COMTval158met polymorphism. Further, we can exclude the possibility that this differential treatment response may be driven by the met/met group having tendentially lower anxiety symptom scores prior to treatment and thus less room for improvement, as we inclusion of the pre-treatment symptom scores as a covariate in our additional analysis did not change our results.

Importantly, including additional covariates (age, depression, medication status, sex, type of CBT treatment, pre-treatment symptom scores) that may theoretically have an effect on our outcome measures did not change our results significantly. Thus, even though the literature suggests a sex-specific effect of the COMTval158met polymorphism in mental disorders [e.g., [12,20]], we did not detect evidence for a sex-specific effect on symptom ratings or efficacy of CBT treatment.

Exposure treatment in CBT which is a gradual exposure to the actual, feared stimulus in the patients mind and real life situations is the most empirically validated and active ingredient of CBT for anxiety disorders. This procedure is based on the theory that the fear response has been classically conditioned and that subsequently negatively reinforced avoidance behavior maintains this fear [21]. Through exposure to the feared stimuli this vicious circle can be interrupted and extinction occurs as desensitization to the anxiety-provoking stimuli. Extinction represents not simply forgetting but an active learning process resulting in structural and chemical changes in the brain [22].

It is both of theoretical and clinical interest to identify biological markers for individualization of both CBT and pharmacological treatment because a significant proportion of patients do not respond to either one or both treatment forms or do not tolerate and complete a specific treatment.

Currently, there is a lack of studies investigating the role of genetic variants in the efficacy of CBT in PD, while some pharmacogenetic studies exist [e.g. [23]]. This gap in the literature probably originates in the historical bias to consider pharmacological treatment as a "biological" treatment which would have biological correlates of symptoms relief. CBT in turn is historically considered as a psychosocial intervention without biological correlates. However research has clearly shown, that the changes in affect, behavior and cognition seen after CBT treatment have biological underpinnings and are accompanied by significant and disorder specific changes in brain metabolism [for a review see eg., [1,24]].

Despite the paucity of studies in PD, studies concerning other psychiatric disorders are emerging. Very recently, it has been shown in PTSD patients that carriers of the 5-HTTLPR s-allele/L_G _profit less from CBT as compared to non-carriers in a sample of 45 patients [25]. This has not yet been investigated in PD patients and may be an interesting additional or even a confounding factor in our results. Thus, 5-HTTLPR/rs25531 genotype was also analyzed in our material and the major analyses of this manuscript were also performed with 5-HTTLPR/rs25531 genotype as dependent variable (see additional files 2 and 3). While the s-allele/L_G_-allele of the 5-HTTLPR (both bi- and triallelic) genotype was associated with higher symptom severity prior to CBT, no association between this genotype (both bi- and triallelic) and the outcome of CBT was observed. Importantly, the association of COMTval158met genotype remained significant after entering 5-HTTLPR (both bi- and triallelic) as an additional covariate (p = 0.03). Thus 5-HTTLPR seems not to be associated with outcome of CBT treatment in PD patients.

Panic attacks are thought to originate from an abnormally sensitive fear network involving the amygdala [21]. During treatment, the amygdala is thought to be inhibited either directly by medications such as SSRIs or indirectly by medial and ventral prefrontal areas exerting a kind of cognitive control over the amygdala [26]. The latter process is thought to be mediated by CBT, which shares a final common result with pharmacotherapy: namely symptom relief. The COMTval158met polymorphism may have a significant impact on this type of top-down regulation as it is known to affect prefrontal dopamine levels and prefrontal cortex functioning and the emotional perseveration, observed as a failure of extinction or obtaining symptom relieve from exposure-based CBT in the met/met group, most likely reflects impaired cognitive control over emotional reactions.

Prior studies investigating the COMTval158met polymorphism in PD have mainly focused on case-control comparisons and results have been mixed. Our finding of (tendentially) higher symptom severity in val-allele carriers prior to CBT treatment corroborates several findings on an association of the val-allele with PD [10,11]. A recent meta-analysis [12] suggested that in Caucasians the val-allele may be associated with PD, while in Asians the met-allele may be the risk allele for the development of PD. Our data suggest that the COMT 158val allele may be associated with more severe symptoms of general anxiety and possibly also depression in PD patients.

On the other hand in our dataset, patients with the met/met genotype seem to be overrepresented. It is known that a deviation from HWE could also reveal possible genotyping problems. However we used a Taqman^® ^assay for genotyping which is a very robust method and all the genotypes were determined in duplicates. We also run the samples on the same 384-plate as samples from other to date unpublished studies involving healthy individuals and these populations did not show a deviation from HWE. As genotyping error seems unlikely, patients with the met/met genotype seem to be overrepresented among our PD patients. As we do not report data on an additional healthy control group, we cannot draw any conclusions about overrepresentation of these individuals. Future studies using much larger sample sizes as well as adequate control groups should clarify if individuals with the met/met genotype are overrepresented in patients with PD and thus may be more prone to the development of PD.

### Limitations

Limitations of our study include: First, the use of the HADS can be questioned as it is not a direct measure of either panic or depression, but rather symptoms of general anxiety symptoms (anxiety subscale) and the state of anhedonia (depression subscale) [15]. However our findings on the effect of the COMTval158met genotype on CBT treatment outcome were specific for the anxiety subscale, indicating that the symptom reduction of general anxiety symptoms specifically are associated with the COMTval158met genotype. Nevertheless, future studies should make sure to also include panic-specific symptom dimensions in addition to general anxiety and depression.

Second, our sample may be biased by a selection on social class and level of education as access to and willingness to use internet was a prerequisite for inclusion, thus limiting the generalizability of our findings. On the other hand, the majority 92%) of Swedish citizens has regular access to the Internet http://www.internetworldstats.com.

Third, it needs to be mentioned that almost half of the patients were medicated and it is possible that there may be an overrepresentation of patients who did not respond well to the pharmacological treatment. We cannot finally exclude the possibility that medication status may affect treatment outcome, maybe even in a genotype dependent manner or that medication may have additional effects during the observational period even after 8 weeks of stable medication. However an effect of co-medication is rather unlikely, given that we do not find an effect of medication status on the symptomatic profile at any point of time. No differences in medication status between the genotype groups and treatment arm groups were observed either. Furthermore and importantly, entering medication status as a covariate in our primary analyses still yielded a significant effect of COMTval158met genotype on outcome of exposure based CBT treatment.

Fourth, we cannot rule out the possibility of a differential efficacy of one of the two treatment modes (iCBT vs. gCBT) depending on the COMTval158 genotype. However the frequency of the COMTval158met genotype groups did not differ between the two treatment arms (see Table 1) and entering type of treatment as an additional covariate in our analysis did not change our results significantly. Additionally and importantly, it has been demonstrated convincingly that iCBT and gCBT do not differ in their efficacy in the treatment of PD [27-29,13]. Nevertheless future studies using larger sample sizes, only non-medicated patients and more homogeneous treatment protocols are warranted to follow-up on our findings.

Fifth, given that we observed pre-existing differences with respect to the frequency of mild co-morbid depression this may have introduced a bias in our results. This difference when it comes to the frequency of co-morbid depression may in fact be meaningful in itself and in this case, controlling statistically for this differences would be inappropriate. However and importantly, we demonstrated in our additional analyses that our primary results do not differ when including or not including this variable as a covariate in the analyses.

Sixth, due to missing HADS measurements after each module, we were not able to perform a repeated measurement ANOVA and merged modules with similar content to blocks (cognitive vs. exposure block). The reason for the missing data, in particular in later modules, is that the number of modules patients went though during the ten-week treatment period differed between patients. Importantly, the number of modules finished did not differ between the COMTval158met genotype groups (see Table 1). Thus it is highly unlikely that our results are due to a selective drop out of patients moving forward slowly.

Seventh, we cannot exclude the possibility that the differential effect during the exposure block seen for the COMTval158met genotype groups may in fact represent an effect of the cognitive modules or treatment generally with a delayed onset or a more consolidated fear memory rather than an effect of extinction. Treatment studies have however shown that for CBT for phobic disorders, behavioral interventions (relying heavily on the principle of extinction) predominate over cognitive ones [for a review see e.g., [24]]. In light of this and in particular given the theoretical basis of our hypothesis we consider it anyhow justified to interpret our results suggestive of a differential effect of exposure based treatment methods.

Interestingly, treatment outcome to different types of treatments has also been associated to the COMTval158met polymorphism in various patient populations. In depressive patients the val/val genotype has been associated with better response to electroconvulsive treatment [ECT, [30,31]][32] and pharmacological treatment [33,34]. However, it needs to be mentioned that the literature is not entirely conclusive [35]. In addition, treatment resistant schizophrenics with the met/met genotype required higher doses of antipsychotic medication to achieve a significant reduction in psychotic symptoms [36]. Thus, patients with the met/met genotype may be generally less responsive to any form of treatment as compared to their val/val counterparts and their brain may be less plastic to changes induced by interventions.

## Conclusions

Despite of these limitations, this is to our knowledge the first study in which the response to CBT has been associated with a genetic marker in PD. The field of pharmacogenetics has in principle been limited to the study of drugs while CBT has only been considered in one very recent report in PTSD [37]. Additionally, PD has been fairly neglected so far in pharmaco/psychotherapy-genetic studies. The clinical implication of our finding is that the outcome of psychological treatments may be gated by the patient's genetic profile. If the patient's genetic profile can be related to the likelihood to profit from a specific form of treatment, this would imply that treatment could be optimized according to this profile. While the development of drugs that may target a small population is quite costly, personalized administration of CBT would not be likewise cost-intensive. It may be possible that patients with the met/met genotype would need a more intense or prolonged exposure treatment in order to achieve the same symptom relief as patients that carry a COMT val -allele. Alternatively they may profit from additional treatment (e.g. pharmacotherapy). In fact, cognitive enhancers such as d-cycloserine have been found to boost the effect of exposure treatment [38]. It is possible that this effect may be gated by the patients genetic make-up. Similar to drug dosage recommendations, the intensity or dosage of CBT may take the genetic profile of the patient into account.

Psychotherapy can be regarded as a form of learning from the environment, and the learning process occurring during psychotherapy most likely produces alterations of gene expression and thereby strength in synaptic connections in the brain [39]. That we find a common genetic polymorphism (COMTval158met) to be differentially associated with the efficacy of exposure-based treatment in panic patients may suggest an interesting new possible venue of gene × environment interaction, even though the association study approach that we used does not allow to conclude a causal link and future replication studies using larger samples (300-500 individuals) are however warranted.

## Competing interests

The authors declare that they have no competing interests.

## Authors' contributions

Conceived and designed the study: TBL CR JB MS AÖ NL. Carried out the genetic experiments: TBL MS. Responsible for the psychological treatment JB, GA. Responsible for the diagnostic procedures: CR NL. Analyzed the data: TBL JB GA CR. Contributed reagents/materials/analysis tools: TBL MS. Wrote the paper: TBL, CR. All authors critically revised, read and approved the final manuscript.

## Pre-publication history

The pre-publication history for this paper can be accessed here:

http://www.biomedcentral.com/1471-244X/10/99/prepub

## Supplementary Material

Additional file 1 Figure S1: Flow chart patient inclusion. Click here for file

Additional file 2 **Additional Methods: Genotyping: 5-HTTLPR/rs25531**. Provides additional genotyping methods. Click here for file

Additional file 3 **Additional Analyses 2: 5-HTTLPR**. Provides the same analyses as in the main manuscript for bi and triallelic 5-HTTLPR. Click here for file

Additional file 4 **Additional Analyses 1: COMTval158met (val/val, val/met, met/met)**. Provides the same analyses as in the main manuscript for the three COMTval158met genotypes (as opposed so a-priori merging carriers of one or two val-alleles). Click here for file
