# Supplementary figures and images for: The COMTval158met polymorphism is associated with symptom relief during exposure-based cognitive-behavioral treatment in panic disorder
Source: BMC Psychiatry. 2010 Nov 26;10:99. doi: 10.1186/1471-244X-10-99 (PMC3004861; doi:10.1186/1471-244X-10-99)

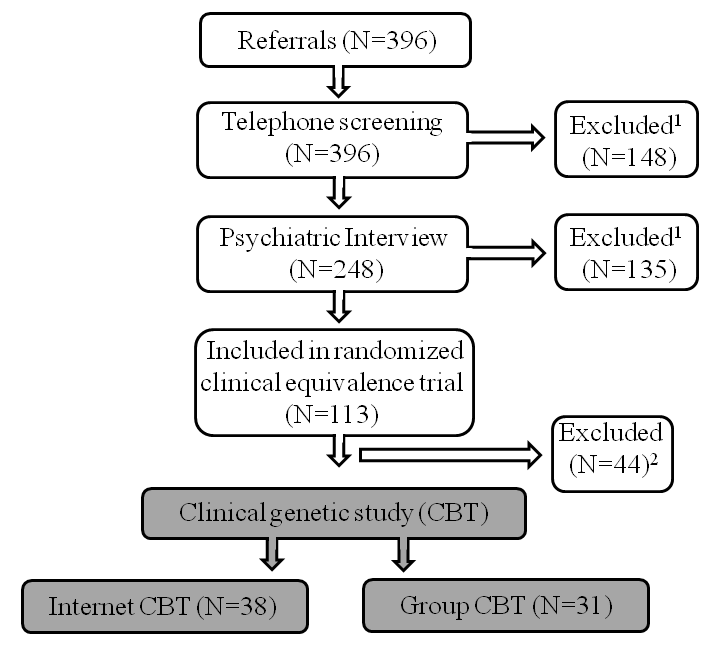

Supplement: Additional file 1 — Figure S1: Flow chart patient inclusion. [file 1471-244X-10-99-S1.TIFF]
