# Supplementary material for: The COMTval158met polymorphism is associated with symptom relief during exposure-based cognitive-behavioral treatment in panic disorder
Source: BMC Psychiatry. 2010 Nov 26;10:99. doi: 10.1186/1471-244X-10-99 (PMC3004861; doi:10.1186/1471-244X-10-99)
Supplement: Additional file 2 — Additional Methods: Genotyping: 5-HTTLPR/rs25531. Provides additional genotyping methods. [file 1471-244X-10-99-S2.DOCX]

**Additional Methods: Genotyping: 5-HTTLPR/rs25531**

DNA was extracted from whole blood as described by Geijer et al. (Geijer *et al.*, 1994). Genotyping for 5-HTTLPR was performed as described in detail earlier [1]. Briefly, for the biallelic 5-HTTLPR two fragments, 336b (short) and 379bp (long), were amplified by polymerase chain reaction (PCR), separated for 2h at 180 V by gel8 electrophoresis in TBE-buffer on a 2.5% Agarose gel containing ethidium bromide for ca. 2h and visualized using ultraviolet light (UV). All genotypes were determined in duplicates. We used the modified nucleotide 7-deaza-dGTP in our initial protocol and could not obtain a satisfying digestion with either MSPI or HPAII enzymes to determine the triallelic 5- HTTLPR. This problem has been reported earlier [2] and therefore a different protocol was used to determine the rs25531 genotype.

For the triallelic 5-HTTLPR, PCR reactions were performed in a final volume of 20μl containing 50ng of genomic template, 0.2mM each deoxynucleoside triphosphate (dNTP), 1mM each primer, 0.05U/μl Quiagen HotStar®Polymerase, 1M Q-Solution and 1x Buffer. The primer (Thermo Scientific, Ulm, Germany) sequences were Forward: 5'- GGCGTTGCCGCTCTGAATGC-3' and Reverse. 5'-GAGGGACTGAGCTGGACAACCAC- 3'. Samples were amplified on a Biorad Tetrade (BIORAD, Hercules, CA, USA) with an initial denaturation step for 10min at 94°C followed by 32 cycles consisting of denaturation for 30s at 95°C, annealing for 30s at 57°C and elongation for 30s at 72°C and one final elongation step for 5min at 72°C. This yields a 486bp (short) and a 529bp (long) fragment. 8

μl of the PCR product separated for 2h at 180 V on a 2.5% agarose gel containing GelRed® and visualized using UV light. 10μl of the remaining PCR product were digested for 12h at 37°C with 0.1μl MSP1 (New England Biolabs, Ipswich, MA, USA) and 1μl buffer per sample. MSP1 recognizes and cuts a 5’-C/CGG-3’ sequence resulting in the following fragments: 340bp, 127bp and 62bp for the LA allele, 297bp, 127bp and 62bp for the SA allele, 174, 166, 127 and 62bp for the LG allele and 166, 131, 127 and 62bp for the SG allele.

Fragments were run for 2h at 180 V on 4% Agarose gels containing GelRed® and visualized via UV light. All biallelic 5-HTTLPR genotypes were thus determined using two different protocols that yielded identical results.

The 6 observed genotypes of the triallelic 5-HTTLPR (5-HTTLPR/rs25531) were grouped based on assumed expression [3] into high (LA/LA), intermediate (LA/LG, SA/LA) and low expression (SA/LG, SG/LG andSA/SA).

**Additional References**

1. Lonsdorf TB, Weike AI, Nikamo P, Schalling M, Hamm AO, Ohman A: **Genetic Gating of Human Fear Learning and Extinction: Possible Implications for Gene-Environment Interaction in Anxiety Disorder.** *Psychological Science* 2009, **20:**198-206.

2. Grime SK, Martin RL, Holaway BL: **INHIBITION OF RESTRICTION ENZYME CLEAVAGE OF DNA MODIFIED WITH 7-DEAZA-DGTP.** *Nucleic Acids Research* 1991, **19:**2791-2791.

3. Kraft JB, Slager SL, McGrath PJ, Hamilton SP: **Sequence analysis of the serotonin transporter and associations with antidepressant response.** *Biological Psychiatry* 2005, **58:**374-381.

4. Lonsdorf TB, Rück C, Bergström J, Andersson G, Öhman A, Schalling M, Lindefors N: **The symptomatic profile of panic disorder is shaped by the 5-HTTLPR polymorphism.** *Progress in Neuro-Psychopharmacology and Biological Psychiatry* 2009, **33:**1479-1483.
