# Supplementary material for: The COMTval158met polymorphism is associated with symptom relief during exposure-based cognitive-behavioral treatment in panic disorder
Source: BMC Psychiatry. 2010 Nov 26;10:99. doi: 10.1186/1471-244X-10-99 (PMC3004861; doi:10.1186/1471-244X-10-99)
Supplement: Additional file 3 — Additional Analyses 2: 5-HTTLPR. Provides the same analyses as in the main manuscript for bi and triallelic 5-HTTLPR. [file 1471-244X-10-99-S3.DOCX]

**Additional Analyses 2: 5-HTTLPR**

*Patient characteristics and genotype frequencies*

We observed the following bi-allelic 5-HTTLPR genotypes in our dataset: N=9 s/s, N=38 s/l, N=22 l/l and for the so-called “triallelic 5-HTTLPR” (5-HTTLPR/rs25531) we observed the following genotypes as grouped according to the classification by Bryant et al. [1] (N=46 S/L_G_-carrier includes S_A_S_A_, S_A_L_A_, S_A_L_G_, S_G_L_G_, L_A_L_G_ and L_G_L_G_ genotypes vs. N=13 L_A_L_A_ genotype).

S-carriers and non-carriers did not differ in any of the clinical variables (comorbid mild major depressive episode, medication, sex age, duration of illness, age of onset, assignment to iCBT vs gCBT, number of modules), all F<1.

For the analyses performed to compare our data with the data of Bryant, sex differed significantly between the genotype groups and thus was included as a covariate in the relevant analyses, p=0.023 (N=8 L_A_L_A_ male, N=5 L_A_L_A_ female, N= 18 s-carrier male, N=38 s-carrier female).

*Pre-treatment*

Both depressive and anxiety symptom severity (as measured by the HADS) prior to CBT were associated with 5-HTTLPR genotype when comparing s-carriers with non-carriers (l/l), when comparing all three genotypes of the biallelic 5-HTTLPR (s/s, s/l, l/l) as well as the triallelic 5-HTTLPR (see additional Table 1). S-carriers/L_G_-carriers reported more symptoms.

**Additional Table 1**

| HADS Anxiety Subscale | | | | | |
| --- | --- | --- | --- | --- | --- |
|  | | df | F | p | Eta^2^ |
| 5-HTTLPR_s^*1^ | | 1,64 | 6.86 | 0.01 | 0.10 |
| 5-HTTLPR ^*2^ | | 1,63 | 3.38 | 0.04 | 0.10 |
| Triallelic 5-HTTLPR ^*3^ | | 1,65 | 6.40 | 0.01 | 0.09 |
| HADS Depression Subscale | | | | | |
|  | df | | F | p | Eta^2^ |
| 5-HTTLPR_s^*1^ | 1,64 | | 8.55 | 0.005 | 0.12 |
| 5-HTTLPR ^*2^ | 1,63 | | 7.48 | 0.001 | 0.19 |
| Triallelic 5-HTTLPR ^*3^ | 1,65 | | 6.97 | 0.010 | 0.10 |

^*1^ SERT_s = s-carriers vs. non-carriers

^*2^ SERT = s/s, s/l, l/l

^*3^ Triallelic SERT_s = LA/LA vs. s-allele/LG-allele carrier (LA/LG, SA/LA, SA/LG, SG/LG andSA/SA)

*Efficacy of CBT*

We do not find any association of the 5-HTTLPR with the efficacy of CBT (for pre treatment – cognitive block and cognitive-exposure block) as measured by the anxiety and depression scale of the HADS in PD patients when comparing s-carriers to non-carriers (l/l), when comparing all three genotypes of the biallelic 5-HTTLPR (s/s, s/l, l/l) or when using the triallelic 5-HTTLPR classification (see above), all p>0.1. Controlling for pre-treatment symptom scores did not alter these results.

*Additional Analyses – ( long-term) efficacy of CBT*

In order to assure maximal comparability with the sudy by Bryant et al. [1], we also analyzed our data in a way maximally similar to the analyses described in their article.

Because HADS measurements were only available *during* the course of treatment but not for a six-month follow up measurement we selected a different instrument for these analyses. Panic Disorder Severity Scale (PDSS, [2]) ratings were available for the measurements prior to treatment, after treatment as well as for the six month follow-up measurement (but not *during* the course of treatment).

We performed a repeated measurements ANOVA (N=54 whereof 11 La/La and 36 s-carrier/Lg-carrier) using number of modules went through as a covariate. We find a significant effect of measurement period (with symptom severity decreasing over time), F(2,100)=6.25, p=0.03, Eta^2^=0.11, but no interaction with genotype F(2,100)<1. However we find a main effect of genotype, F(1,50)=6.34, p=0.015, Eta2=0.11, with carriers of the s-allele and/or l_G_-allele reporting significantly more symptoms.

Even though we do not find any indication of an interaction of measurement period (time) with genotype, we explicitly tested for possible genotype differences for the follow-up measurement using a univariate ANOVA (controlling for number of modules went through, sex and pre-treatment PDSS score) as Bryant et al., find significant differences between 5-HTTLPR s-carriers and non-carriers only for this measurement period. In our dataset, we do however not find a significant association between 5-HTTLPR genotype and long-term treatment outcome of CBT in PD patients, F(1,53)<1.

1. Bryant RA, Felmingham KL, Falconer EM, Benito LP, Dobson-Stone C, Pierce KD, Schofield PR: **Preliminary Evidence of the Short Allele of the Serotonin Transporter Gene Predicting Poor Response to Cognitive Behavior Therapy in Posttraumatic Stress Disorder.** *Biological Psychiatry* 2010, **67:**1217-1219.

2. Shear MK, Brown TA, Barlow DH, Money R, Sholomskas DE, Woods SW, Gorman JM, Papp LA: **Multicenter collaborative panic disorder severity scale.** *Am J Psychiatry* 1997, **154:**1571-1575.
