# Supplementary material for: The COMTval158met polymorphism is associated with symptom relief during exposure-based cognitive-behavioral treatment in panic disorder
Source: BMC Psychiatry. 2010 Nov 26;10:99. doi: 10.1186/1471-244X-10-99 (PMC3004861; doi:10.1186/1471-244X-10-99)
Supplement: Additional file 4 — Additional Analyses 1: COMTval158met (val/val, val/met, met/met). Provides the same analyses as in the main manuscript for the three COMTval158met genotypes (as opposed so a-priori merging carriers of one or two val-alleles). [file 1471-244X-10-99-S4.DOCX]

**Additional Analyses 1: COMTval158met (val/val, val/met, met/met)**

In our manuscript, carriers of one or two COMT 158val-alleles (val/val and val/met) were *a-priori* pooled and compared to individuals with the met/met genotype. This strategy is often used to increase statistical power and was in our case also based on prior data and a specific hypothesis [1]. However, pooling two genotype groups leads to loss of data and may hamper the ability to compare different studies (e.g. for metaanalyses). Therefore, we include the main analyses of our manuscript comparing all three COMTval158met genotype groups (met/met, val/met, val/val) as additional material the main analyses.

*Symptomatic profile prior to CBT treatment*

Prior to treatment, heterozygous patients (val/met) reported more symptoms as compared to patients with the met/met genotype in both the anxiety subscale of the HADS, F(2,63)=4.39, p=0.002, pη^2^=0.12, and the depression subscale of the HADS F(2,63)=2.64, p=0.08, pη^2^=0.08. Further analyses revealed that heterozygous reported more anxiety symptoms as compared to both patients homozygous for the (met) met/met, p=0.006 and the val (val/val) allele, p=0.035. For the depression subscale, homozygous for the met allele (met/met) reported significantly less symptoms then patients homozygous for the val allele (val/val), P=0.026. Heterozygous did not differ significantly from either patients with the met/met, p=0.48 or the val/val genotype, p=0.14.

*Symptomatic profile after treatment*

No significant differences between the COMTval158met genotype groups on HAD anxiety or depression subscale were found after treatment.

*Symptom relief during the course of treatment*

*Difference score pre-treatment – cognitive block*

An univariate ANOVA with the difference score between the mean HADS anxiety score prior to treatment and during the cognitive block as the dependent and COMT158met genotype as the independent variable revealed no significant effect of COMTval158met genotype on this difference score, F(2,59)<1.

No effect was found for the HADS depression scale during the course of treatment using the same analysis as described above either, F(2,59)<1.

*Difference score cognitive block – exposure block*

A univariate ANOVA with the difference score between the mean HADS anxiety score of the cognitive and the mean HADS anxiety score of the expose block as the dependent, COMT158met genotype (met/met, val/met, val/val) as the independent variable revealed a significant effect of COMTval158met genotype, F(2,55)=2.87, p=0.071, pη^2^=0.09. Individuals with the met/met genotype showed significantly less symptom relief during the exposure block as compared to individuals with the val/val genotype, p=0.025 but not heterozygotes (val/met), p=0.11. Carriers of one (val/met) and two val-alleles (val/val) did not differ either, p=0.056.

An additional analysis including medication status (yes/no), sex, pre-treatment HADS anxiety score and the type of treatment (ICBT vs. gCBT) as additional covariates (in addition to depression and age) in the analysis on the difference score between the cognitive and the exposure block revealed no significant impact of these variables. Importantly, the effect of COMTval158met genotype on the outcome measure still remained a tendency when controlling for all these variables, F(2,51)=2.80, p=0.07, pη^2^=0.1.

However, sex had a close to significant impact on the difference score between the cognitive and the exposure block, F(1,51)=3.86, p=0.055, pη^2^=0.07. Women had higher difference scores and thus more symptoms relieve than men. Importantly, additional analyses did not yield evidence for a sex x COMTval158met genotype interaction on the difference score between the cognitive and the exposure block, F<1.

No effect of COMTval158met genotype was found for the HADS depression scale during the course of treatment using the same analyses as

described above, all F<1.1.
